# Multi-Physics Modeling of Melting-Solidification Characteristics in Laser Powder Bed Fusion Process of 316L Stainless Steel

**DOI:** 10.3390/ma17040946

**Published:** 2024-02-18

**Authors:** Xiuyang Shan, Zhenggao Pan, Mengdi Gao, Lu Han, Joon-Phil Choi, Haining Zhang

**Affiliations:** 1 School of Mechanical and Electronic Engineering, Suzhou University, Suzhou 234000, China; shanxiuyang@ahszu.edu.cn (X.S.);; 2 School of Information Engineering, Suzhou University, Suzhou 234000, China; 3 Department of 3D Printing, Korea Institute of Machinery & Materials, Daejeon 34103, Republic of Korea; 4 School of Mechanical and Aerospace, Nanyang Technological University, Singapore 639798, Singapore

**Keywords:** laser powder bed fusion, finite volume method, 316L stainless steel, temperature distribution, keyhole depth

## Abstract

In the laser powder bed fusion process, the melting-solidification characteristics of 316L stainless steel have a great effect on the workpiece quality. In this paper, a multi-physics model was constructed using the finite volume method (FVM) to simulate the melting-solidification process of a 316L powder bed via laser powder bed fusion. In this physical model, the phase change process, the influence of temperature gradient on surface tension of molten pool, and the influence of recoil pressure caused by the metal vapor on molten pool surface were considered. Using this model, the effects of laser scanning speed, hatch space, and laser power on temperature distribution, keyhole depth, and workpiece quality were studied. This study can be used to guide the optimization of process parameters, which is beneficial to the improvement of workpiece quality.

## 1. Introduction

Under the assistance of additive manufacturing (AM) technology, periodic or statistically homogeneous materials can be used to explore a wider range of interesting structures [1]. As a part of the AM technology, laser powder bed fusion (LPBF) is widely used in the manufacturing of metallic material. By using high temperature restricted to local areas, this technology can directly manufacture the metallic powder into any part geometries or complex specimens with some inner degrees of freedom with a layer-by-layer fashion [2,3,4,5]. However, this results in a large temperature gradient, which has great effects on thermal stress. During LPBF, the process parameters have great effects on heat transfer characteristics and molten pool morphology in the melting-solidification process of the powder bed [6,7]. The inappropriate selection of process parameters may also result in undesirable effects, such as thermal cracking, balling, and porosity [8].

The 316L stainless steel (SS316L) has been widely used in navigation, nuclear reactor, aerospace, and medical environments for many years due to its perfect mechanical properties, such as high tensile strength, low coefficient of thermal expansion, perfect resistance to oxidation at high temperature, creep resistance, and fatigue resistance [9,10,11]. In recent years, the SS316L powder has been widely used in AM to create complex shaped structures due to the properties of excellent corrosion resistance, low carbon content, high-temperature resistance, weldability, and good flowability of the powder bed, which makes it well-suited for AM. Moreover, research shows that the nearly full densification part of SS316L can be achieved using the LPBF process [12]. While SS316L offers numerous advantages in AM, there are also some limitations and challenges associated with its use in AM, such as high thermal conductivity, which makes it challenging to achieve optimal melt pool; residual thermal stresses and distortion, which may potentially cause warping or distortion; and inhomogeneous microstructure and porosity, which may affect the mechanical properties. To obtain high-performance final parts of SS316L manufactured by the LPBF process, current research interest in LPBF primarily focuses on optimizing process parameters, analyzing microstructures, characterizing mechanical properties, exploring post-processing techniques, and employing computational modeling and simulation. This collective research effort aims to advance our understanding and enhance the quality of SS316L parts produced via LPBF [13,14,15,16,17,18]. 

During the LPBF process, the SS316L powder bed is rapidly melted, solidified, and even vaporized. The flow behavior in the molten pool can be affected by these phenomena. Thus, the thermal distribution and molten pool morphology will be changed. To analyze the relationship between the LPBF process and the mechanical properties of final parts, some experimental and theoretical research has been conducted by other scholars. Greco et al. conducted LPBF experiments of SS316L powder with constant laser energy density. Via these experiments, the effects of laser power, layer thickness, and hatch space on workpieces’ roughness, density, and micro-hardness were studied [19]. Röttger et al. manufactured some specimens using SS316L with different types of LPBF devices. Meanwhile, their microstructures were observed by means of scanning electron microscopy. It was found that these specimens possessed similar microstructures, but there was different porosity between the microstructures, which makes the specimens have significant differences in mechanical strength [20]. Suryawanshi et al. compared the part of SS316L produced using LPBF with the conventionally manufactured austenitic stainless steel. By observing their micro and meso-structures, the property variation was analyzed [21]. Şener et al. conducted a series of experiments to study the properties of the samples by changing the process parameters. Via optical profilometry and tensile test, the effects of process parameters on mechanical performance and surface quality were obtained and optimized [22]. The above experiments can efficiently analyze the mechanical properties of the final parts processed by LPBF, but the effect of the melting-solidification mechanism of the metallic powder bed is hard to analyze for the characteristics of molten pool, such as large temperature gradient and flow behavior at a small scale.

Considering the molten pool characteristics, the mechanism model is suitable to analyze the melting-solidification process caused by the rapid temperature change in the LPBF process. The developed numerical model of LPBF by other scholars can be used to guide the improvement of the mechanism model for SS316L. Waqar et al. developed an FE model of LPBF to analyze the influence of process parameters on thermal and stress profile [23]. Trejos et al. also developed an FE model of LPBF, which takes into account most of the manufacturing parameters and thermophysical properties. Using this model, the thermal history of the LPBF process was calculated. By considering nodes with temperatures higher than the melting point as molten status, the evolution of the molten pool dimensions was analyzed [24]. Luo et al. developed a new efficient and accurate FE model for predicting transient material thermal behavior during the LPBF process in part-level. To reduce the computational cost, the adaptive mesh scheme was used, which makes the simulation speed 12× to 18× faster compared to the traditional simulation scheme [25]. As the liquid metal flow behavior was not considered in the above models, it is hard to describe the heat transfer process caused by liquid metal flow. Additionally, due to the wide application of LPBF in industrial production, CAE software (ANSYS version 2019R1) providers also developed solutions to simulate the LPBF process. These solutions can accurately analyze the residual deformations and internal stresses under large temperature gradients [26,27,28], but the inhomogeneity of the powder bed distribution and the liquid metal flow make it hard analyze their effects on the melting-solidification process. To analyze the melting-solidification process of SS316L thoroughly, a comprehensive model coupling heat transfer physics and laminar flow physics is eagerly needed.

In this study, a three-dimensional numerical model of LPBF for SS316L was developed to describe the temperature and velocity field in the melting-solidification process. Via the single and multi-track simulation, the effects of process parameters on temperature distribution, keyhole depth, and workpiece quality were studied. By shedding light on the characteristics of the melting-solidification process, the correlation with the process parameters can be clarified. Based on this study, some phenomena during manufacturing can be explained. Meanwhile, it may contribute to the design of experiments when analyzing the correlation between the process parameters and mechanical properties of 316L stainless steel produced by LPBF.

## 2. Computational Models

**Figure 1:**
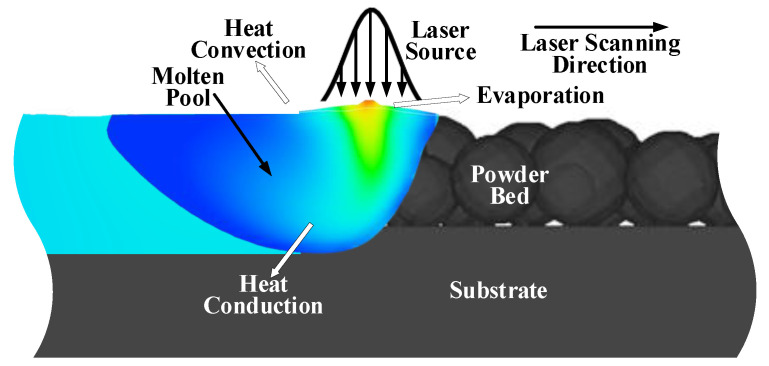
Schematic diagram of the LPBF process. The gray represents the unprocessed steel; the blue represents the molten pool; and the baby blue represents the molten pool after solidification.

During the LPBF process, complex physical phenomena such as heat transfer, phase change, and fluid flow of liquid metal materials are involved, as shown in Figure 1 [29]. By describing the physical phenomena through the corresponding numerical model, the LPBF process can be quantitatively analyzed, which is of great significance to guide the optimization of workpiece quality.

To analyze the critical physical properties in LPBF, the following assumptions should be made to simplify the coupling physical phenomena.

1. The fluid in the molten pool is assumed to be Newtonian and laminar flow;
2. The mushy zone is assumed to be isotropic during the solid–liquid phase change process;
3. The distribution of powder particles is assumed to be fixed in space;
4. The effect of the surrounding argon’s flow on the molten pool is neglected;
5. The influence of ambient gas on laser energy absorption is neglected.

### 2.1. Governing Equations

The mass conservation equation in LPBF is as follows [30]:

(1) $$\frac{\partial \rho }{\partial t}+\nabla \cdot \left(\rho \vec{u}\right)=0$$

where $\vec{u}$ is the velocity of liquid metal; *ρ* is metal density.

For the non-uniform density distribution in the molten pool caused by thermal expansion, the buoyancy effects should be considered. Thus, the momentum conservation equation can be described as follows [31]:

(2) $$\rho \frac{\partial \vec{u}}{\partial t}+\rho \left(\vec{u}\cdot \nabla \right)\vec{u}=\nabla \cdot \left(\mu \nabla \vec{u}\right)-\nabla p+\rho \vec{g}+\vec{F_{B}}-\vec{F_{m}}$$

where $\vec{F_{B}}$ is buoyancy described using the Boussinesq approximation $\vec{F_{B}}=\rho \vec{g}\beta \left(T-T_{0}\right)$; *β* is the thermal expansion coefficient; $\vec{F_{m}}$ is used to characterize the difference of fluidity caused by the liquid–solid transition, which is calculated as $\vec{F_{m}}=D\vec{u}$; D is the Darcy drag force term caused by the solidification and can be described as $D=CF_{s}^{2}/\left({\left(1-F_{s}\right)}^{3}+B\right)$; *F_s_* is the local solid fraction in the computational domain; *C* is a constant that depends on the mushy zone microstructure; *B* is a small constant that is used to avoid *D* being infinity when *Fs* = 1.

The energy conservation equation is given as follows:

(3) $$\frac{\partial }{\partial t}\left(\rho E\right)+\nabla \cdot \left(\rho \vec{u}E\right)=q+\nabla \cdot \left(k\nabla T\right)$$

where *k* is the thermal conductivity; *q* is the heat source; *E* is the specific internal energy which can be described as:

(4) $$E=cT+\left(1-F_{s}\right)L$$

where *c* is the specific heat; *L* is the latent heat of melting.

To track the free surface of molten pool, the volume of fluid (VOF) method is used, which can be described as:

(5) $$\frac{\partial \gamma }{\partial t}+\nabla \cdot \left(\gamma \vec{u}\right)=0$$

where γ is the volume fraction of liquid metal material in the computation element.

### 2.2. Initial and Boundary Conditions

In LPBF, the thermodynamic behavior at the boundary includes a laser heat source, heat dissipation by evaporation, heat convection, and radiation with the surrounding environment. So the thermal boundary condition and initial condition can be defined as follows [32]:

(6) $$k\frac{\partial T}{\partial n}=q\left(x,y,z,t\right)-\varepsilon_{r}\sigma_{s}\left(T^{4}-T_{0}^{4}\right)-h_{c}\left(T-T_{0}\right)-q_{ev}$$

(7) $$T\left(x,y,z,0\right)=T_{0}$$

where *T*_0_ is the ambient temperature; *n* is the vector along the normal direction of the top surface of the powder bed; *q*(*x*, *y*, *z*, *t*) represents the laser energy distribution; *ε_r_* is the surface radiation coefficient; *σ_s_* is the Stefan–Boltzmann constant (5.67 × 10^−8^ W/(m^2^·K^4^)); *h_c_* is the natural convection heat transfer coefficient; *q_ev_* is the heat taken away as the molten pool evaporation.

Based on the characteristics of laser emission, the laser heat source is assumed to be Gaussian distribution [33,34]. The heat flux of the laser decays exponentially from the center outward, and it is given as:

(8) $$q\left(r\right)=q_{0}e^{-2r^{2}/\theta^{2}}$$

where *q* is the heat flux; *r* is the radial distance from the laser center; *θ* is the laser radius where $q=q_{0}e^{-2}$; *q*_0_ is the laser beam intensity at *r* = 0. Thus, *q*_0_ can be written as:

(9) $$q_{0}=\frac{2P\xi }{\pi \theta^{2}}$$

where *ξ* is the absorptivity of the powder bed; *P* is the laser power. Thus, the heat flux can be rewritten as:

(10) $$q\left(r\right)=\frac{2P\xi }{\pi \theta^{2}}e^{-2r^{2}/\theta^{2}}$$

For high laser energy density, the molten pool temperature will exceed the evaporation temperature. The evaporative heat loss can be written as [35]:

(11) $$q_{ev}=\frac{0.82\Delta H^{*}}{\sqrt{2\pi MRT}}P_{0}e^{\frac{\Delta H^{*}}{R}\left(\frac{1}{T_{v}}-\frac{1}{T}\right)}$$

where *R* is the gas constant; *P*_0_ is atmospheric pressure; ΔH* is the effective evaporation enthalpy; *M* is the molar mass; *T_v_* is the evaporation temperature.

As the evaporation of the molten pool under high laser energy density, the recoil pressure is considered as follows [31]:

(12) $$P_{re}\left(T\right)=0.54P_{0}e^{\frac{L_{e}M}{R}\left(\frac{1}{T_{v}}-\frac{1}{T}\right)}$$

where *L_e_* is the latent heat of evaporation; *T_v_* is the evaporation temperature; *P*_0_ is the atmospheric pressure.

At the molten pool surface, the pressure balance can be described as [36]:

(13) $$P-2\mu \frac{\partial \vec{v_{n}}}{\partial n}=P_{re}-\sigma \left(\frac{1}{R_{1}}+\frac{1}{R_{2}}\right)$$

where *n* is the normal direction of the molten pool surface; *P* is the pressure at the molten pool surface; *μ* is the dynamic viscosity; $\vec{v_{n}}$ is the normal velocity; *R*_1_ and *R*_2_ are the principal radii of molten pool surface curvature. The temperature-dependent surface tension coefficient can be described as $\sigma =\sigma_{0}-\partial \sigma /\partial T\left(T-T_{ref}\right)$.

For the temperature dependence of the surface tension coefficient, the Marangoni effect appears. The corresponding fluid velocity boundary condition at the molten pool surface can be described as:

(14) $$\mu \frac{\partial \vec{v_{t}}}{\partial n}=-\frac{\partial \sigma }{\partial T}\frac{\partial T}{\partial r}$$

where *r* is the tangential direction of the molten pool surface; $\vec{v_{t}}$ is the tangential velocity.

### 2.3. Simulation Cases and Computation

**Figure 2:**
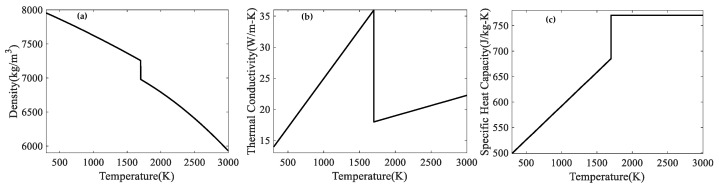
Temperature-dependent parameters of SS316L: (**a**) density [38]; (**b**) thermal conductivity [40]; (**c**) specific heat capacity [40].

The simulation was carried out using the Flow3D V11.1 commercial software to simulate the melting-solidification process and thermal behavior. The simulation domain size is 1000 μm × 400 μm × 250 μm, and the powder layer thickness is 45 μm. The hexahedral mesh elements were used. The mesh size was set to be 5 μm, which is sufficient to describe the non-uniform powder distribution. The corresponding material properties of SS316L are shown in Table 1, and the temperature-dependent parameters are shown in Figure 2.

**Table 1:** Thermal and physical properties of SS316L.

Material Property	Value
Surface tension coefficient	1.76 N/m [37]
Change rate of surface tension	−4.002 × 10^−4^ N/m/K [37]
Density	see Figure 2a
Thermal conductivity	see Figure 2b
Specific heat capacity	see Figure 2c
Solidus temperature	1658 K [38]
Liquidus temperature	1723 K [38]
Evaporation temperature	3086 K [38]
Latent heat of fusion	2.8 × 10^5^ J/kg [10]
Latent heat of evaporation	7.45 × 10^6^ J/kg [38]
Molar mass	5.58 × 10^−2^ kg/mol [38]
Dynamic viscosity	5.6 × 10^−3^ Pa·s [39]
Surface radiation coefficient	0.4 [39]
Absorption coefficient	0.55 (solid), 0.3 (liquid) [10]

## 3. Results and Discussion 

### 3.1. Molten Pool Morphology Analysis

**Figure 3:**
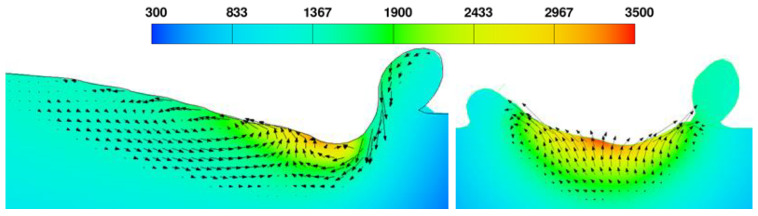
Flow behavior in the molten pool with different cross sections (laser power = 120 W, scanning speed = 0.2 m/s). The black arrows show the fluid flow; the arrow length shows the velocity magnitude; and the color scale shows the temperature field in Kelvin.

When the molten pool temperature exceeded the evaporation temperature, the recoil pressure induced by the escaping vapor of the liquid metal was larger than its surface tension [41]. Thus, the keyhole formed as shown in Figure 3.

At the center of molten pool, the surface temperature was higher than that at the bottom. Thus, the buoyancy appeared due to thermal expansion, resulting in natural convection flowing from the bottom up. The temperature of the molten pool center was also higher than that at the edge. Thus, the surface tension at the molten pool center was smaller than that at the edge for the negative change rate of surface tension. Under the effects of the surface tension, the liquid metal at the center was drawn to the edge [42]. The corresponding flow pattern is shown in Figure 3. This outward flow can promote the bonding of the adjacent scan track after solidification. The asymmetrical circulation in the molten pool was caused by the inhomogeneity of powder distribution, which also created the rough contour line of the scan track after the molten pool solidification.

### 3.2. Temperature Distribution Analysis

**Figure 4:**
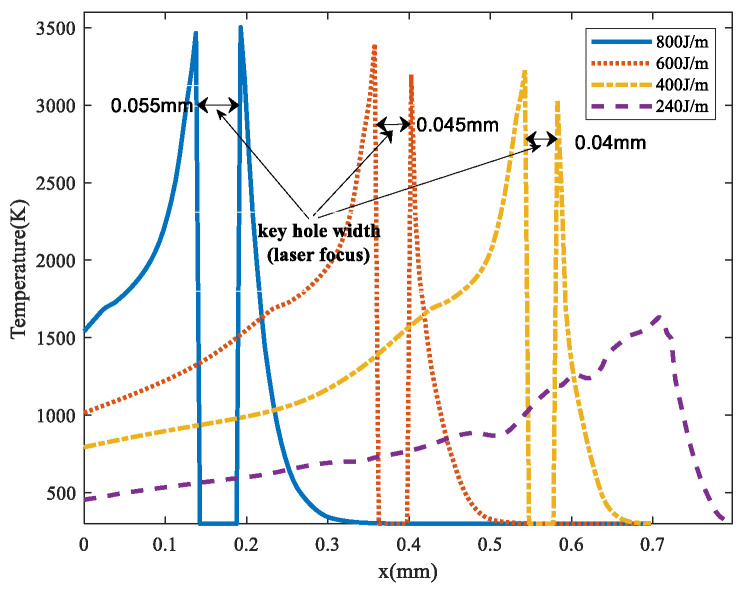
Temperature distribution under different LEDs.

Linear energy density (LED) is commonly used to analyze the temperature distribution in LPBF [43]. Figure 4 shows the temperature distribution on the substrate along the scanning direction under the LED of 240 J/m, 400 J/m, 600 J/m, and 800 J/m respectively, which was obtained at the same laser power of 120 W. It can be found that when the molten pool temperature exceeded the evaporation temperature, the variation of LED had little effect on the temperature near the laser focus. This is because the input energy was taken away by evaporation [41], but the keyhole width increased significantly with the increase of LED for the evaporation enhancement. When the LED decreased to 240 J/m, the recoil pressure on the molten pool surface (Knudsen layer) was wakened, and the keyhole depth decreased significantly, which made it so the laser energy could not be sufficiently transferred to the substrate. Thus, the substrate temperature was lower than the liquidus temperature, which means the previously processed layer and the currently processed one could not be effectively fused in the product manufacturing process, resulting in poor densification [8]. If the LED was further decreased, the densification would drop sharply due to the incomplete melting of the powders [44].

**Figure 5:**
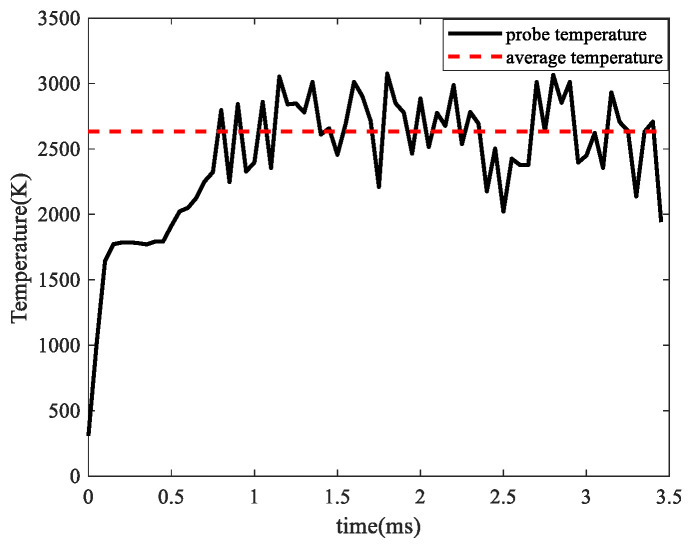
Substrate temperature at laser focus.

To analyze the substrate temperature at laser focus, a temperature sampling probe was set on the substrate, which moved with the laser focus synchronously. To well observe the temperature on the substrate, the keyhole formation on the substrate should be avoided. Thus, the laser power = 120 W and scanning speed = 0.45 m/s were used in this simulation. The temperature at the probe is shown in Figure 5. It could be found that when the laser turned on, the temperature rose quickly. At t = 0.15 ms, the probe temperature reached the liquidus temperature, and the molten pool formed. For the energy absorption of powder melting, the probe temperature of the molten pool rose slowly until the molten pool formed sufficiently. Then, the probe temperature fluctuated around 2635 K (dashed line) due to the non-uniform powder distribution.

**Figure 6:**
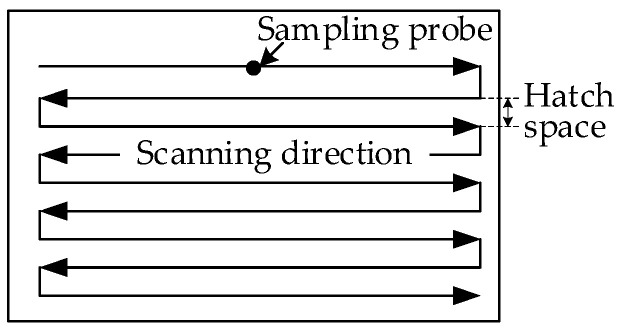
Scanning strategy.

**Figure 7:**
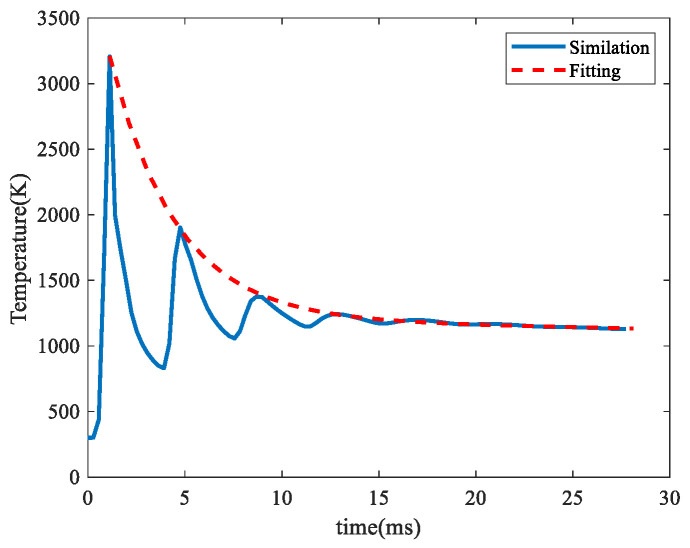
Evolution of substrate temperature at the fixed sampling probe.

Moreover, a fixed temperature sampling probe was set up on the substrate to analyze the multi-track scanning process. In this simulation, the laser power = 120 W, scanning speed = 0.45 m/s, and hatch space = 0.12 mm. The corresponding scanning strategy is shown in Figure 6, and the evolution of temperature is shown in Figure 7. It can be seen that the sampling temperature decreased periodically. In each period, the temperature rose rapidly when the laser focus was close to the sampling probe, and the temperature decreased relatively slowly when the laser focus was far away from the sampling probe. The peak temperature in each period was extracted and fitted as the dashed line in Figure 7, which can be described using the following formula:

(15) $$T\left(t\right)=2777e^{-0.2931t}+1212e^{-0.0024t}$$

where *t*(ms) is laser scanning time. Based on this equation, it can be predicted that the temperature can be reduced to 320 K when the scanning time is 556 ms.

### 3.3. Keyhole Depth Analysis

**Figure 8:**
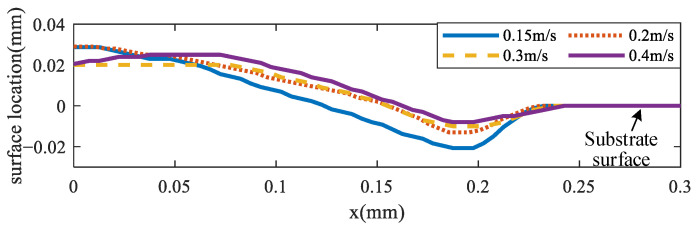
Molten pool surface distribution under different scanning speeds.

**Figure 9:**
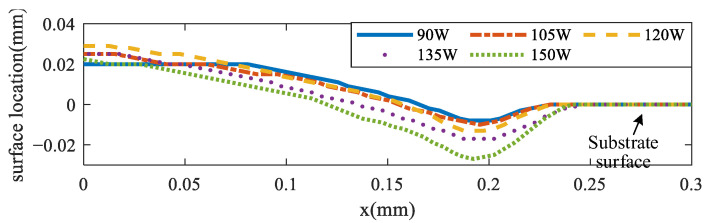
Molten pool surface distribution under different scanning powers.

**Figure 10:**
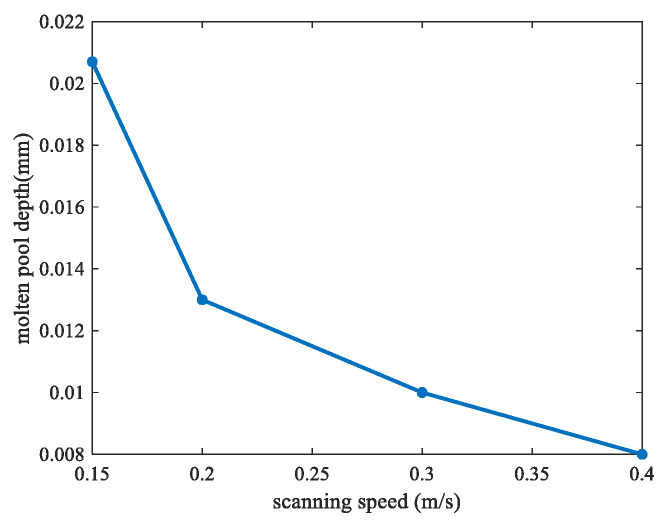
Keyhole depth under different scanning speeds.

**Figure 11:**
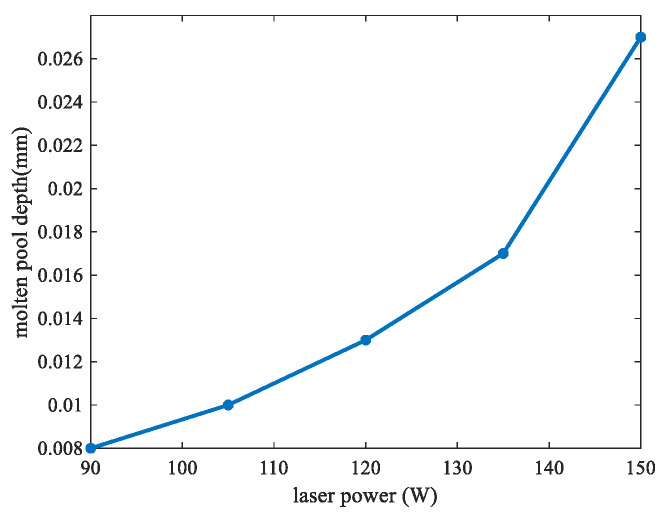
Keyhole depth under different scanning powers.

The keyhole has great effects on the pore formation, which may degrade the performance of the final parts [45]. To simulate the keyhole formation process, different laser scanning speeds and scanning powers were analyzed. Figure 8 shows the molten pool surface distribution for four different scanning speeds (0.15, 0.2, 0.3, and 0.4 m/s) for the same laser power of 120 W, respectively. Figure 9 shows the molten pool surface distribution for five different laser powers (90, 105, 120, 135, and 150 W) for the same scanning speed of 0.2 m/s, respectively. Then, the keyhole depth was obtained, as shown in Figure 10 and Figure 11, respectively. It could be found that with the decrease of scanning speed and the increase of laser power, the gradient of the keyhole depth also increased. This indicates that the keyhole depth can be increased more effectively by increasing the energy input after the keyhole has formed. Moreover, the remelting of the previous track can increase the densification and wetting properties [44].

**Figure 12:**
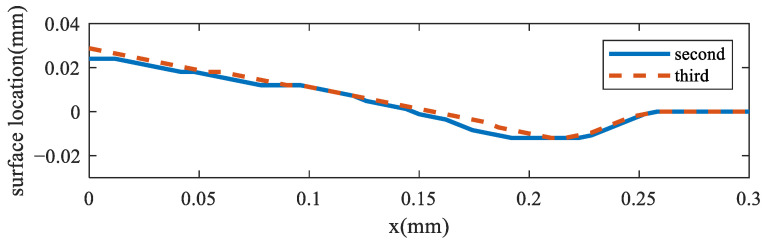
Molten pool surface distribution during multiple-track fabrication.

To analyze the multiple-track interaction, the multiple-track manufacturing process was simulated with laser power = 120 W, scanning speed = 0.2 m/s, and hatch space = 0.12 mm. The molten pool surface distribution of the second and third tracks is shown in Figure 12. It shows that the two molten pools had the same morphology, which means that the residual heat from the previous track had little effect on the keyhole depth of the current one.

### 3.4. Hatch Space Analysis

**Figure 13:**
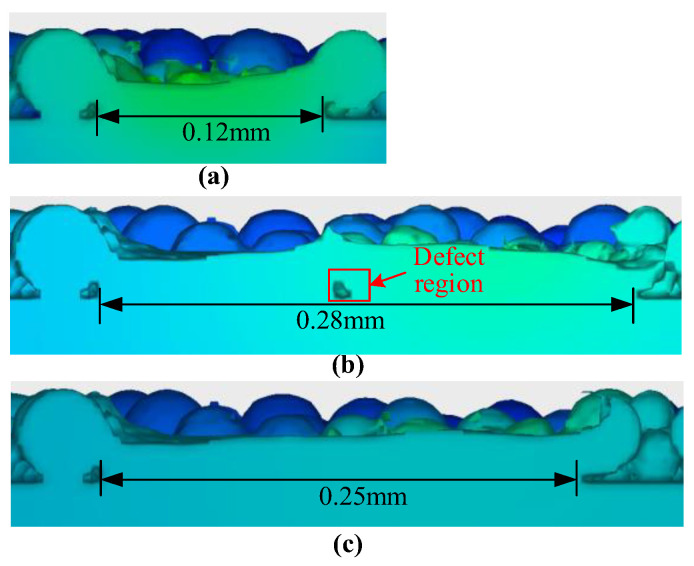
Cross section of molten pool after solidification under different hatch space: (**a**) single-track scanning; (**b**) hatch space = 0.14 mm; (**c**) hatch space = 0.12 mm.

When the laser was 90 W, the scanning speed was 0.2 m/s, the layer thickness was 45 μm, and the stable scan track width was 0.12 mm, as shown in Figure 13a. When the hatch space was set as 0.14 mm, which is larger than the stable scan track width, the internal void defects formed between the adjacent scan tracks, as shown in Figure 13b. To avoid the internal void defects, more overlap of the scan track was needed. Thus, the hatch space was reduced to 0.12 mm, and the stable scan track width was 0.25 mm between adjacent scan tracks, as shown in Figure 13c. By lowering the hatch space, the parts with better density and ultimate tensile strength are manufactured [46].

### 3.5. Analysis of Powder Bed Distribution Effects

**Figure 14:**
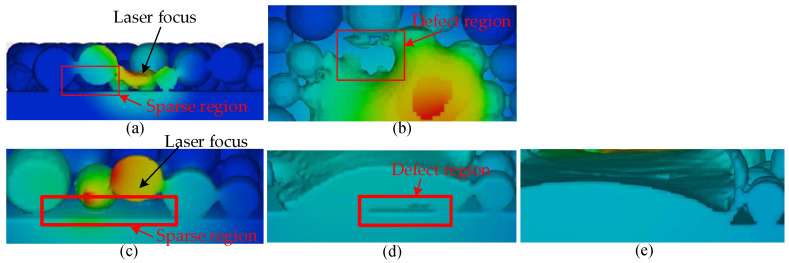
The effects of the powder bed distribution: (**a**) sparse region at the boundary of scan track; (**b**) defect region caused by the sparse region in (**a**); (**c**) sparse region at the center of the scan track; (**d**) defect region caused by the sparse region in (**c**); (**e**) normal morphology of the scan track after increasing the laser power.

When the powders are distributed sparsely in one location, the molten pool morphology will be affected. If the sparse region is located at the boundary of the scan track, as shown in Figure 14a, then the boundary roughness of the solidified region will be worse, as shown in Figure 14b, which is not beneficial to enlarge the scan track width.

If the sparse region is located at the center of the scan track, as shown in Figure 14c, this will result in the internal void defects, as shown in Figure 14d (scanning speed = 0.2 m/s, laser power = 120 W). For such void defects, it can be avoided by increasing the laser power to delay the solidification of the molten pool, which can promote the release of air bubbles in the molten pool, as shown in Figure 14e (scanning speed = 0.2 m/s, laser power = 150 W).

## 4. Conclusions

In this study, the multi-physics simulation model was developed to analyze the melting-solidification process of SS316L in the laser powder bed fusion. Using this model, the temperature distribution and molten pool morphology were analyzed by changing the process parameters. Moreover, the effects of powder distribution on workpiece quality were analyzed. Some valuable conclusions were drawn, as follows:

1. The convection flow in the molten pool can effectively widen the molten pool width and promote strong bonding between adjacent scan tracks. Therefore, the hatch space can be enlarged by increasing the laser power or decreasing the scanning speed to enhance the convection flow behavior.
2. When the LED decreases to 240 J/m, the keyhole depth becomes too small to fuse the previously processed layer with the currently processed one, potentially leading to degradation in part densification. To ensure better densification of the final parts, it is suggested that the LED be set to over 400 J/m when the layer thickness is 45 μm.
3. The keyhole depth can be enlarged more effectively by further increasing the energy input after the keyhole is formed, such as by increasing the laser power or decreasing the scanning speed.
4. If the hatch space is too large or the powder bed is sparsely distributed, internal void defects may form, significantly affecting workpiece quality. To prevent these defects, it is suggested that the hatch space be narrower than the single-track width.

Furthermore, the comprehensive liquid–gas phase change model was not considered for the obstacle of integration with the current simulation model. This may result in the deviation of the temperature field between our numerical simulation and experiments when reaching evaporation temperature. In the follow-up research, a sophisticated simulation model including the liquid–gas phase change should be developed to comprehensively analyze the dynamics of the temperature field after the appearance of evaporation.

## Data Availability

Data are contained within the article.
